# The Burden and Associated Factors of Intestinal Parasitic Infection Among Prisoners in Ethiopia: A Systematic Review and Meta‐Analysis

**DOI:** 10.1155/japr/7271924

**Published:** 2026-09-07

**Authors:** Kemal Mohamed, Abdulhakim Mussema, Asnake Simieneh, Dagmawi Woldesenbet

**Affiliations:** ^1^ Department of Medical Laboratory Sciences, School of Medicine and Health Sciences, Wachemo University, Hossana, Central Ethiopia, Ethiopia, wcu.edu.et; ^2^ Armauer Hansen Research Institute, Addis Ababa, Ethiopia, ahri.gov.et

**Keywords:** Ethiopia, infection, intestinal parasites, prevalence, prisoners, risk factors

## Abstract

**Introduction:**

Intestinal parasitic infection poses a significant health problem among prisoners. This burden arises from inadequate facilities and limited access to health education, malnourishment, limited access to drinkable water, overcrowding, and poor cleanliness.

**Methods:**

PRISMA guidelines were followed in this systematic review and meta‐analysis. To find relevant data, we looked through gray literature sources and published studies through online databases like ScienceDirect, Google Scholar, PubMed, and Semantic Scholar. The Condition, Context, and Population (COCOPO) principle was used to develop search terms. The papers′ quality was evaluated using the critical assessment checklist for prevalence studies developed by the Joanna Briggs Institute (JBI). To arrange, assess, and cite relevant studies, EndNote (Version X9) was utilized. Data analysis was performed using Stata software, Version 17.

**Result:**

This study, included a total of eight studies, involving 2412 prisoners. According to a random‐effects model, the overall prevalence of intestinal parasitic infection among prisoners in Ethiopia was 45% (95% CI: 39%–51%), with a significant degree of heterogeneity (*I* ^2^ = 87.79*%*, *p* < 0.01). In this study, the most common monoinfection was *E. histolytica/dispar* (16%, 95% CI: 12%–23%), whereas the most common multiple infection was *E. histolytica/dispar* + *G. lamblia* (3%, 95% CI: 2%–5%) and *E. histolytica/dispar* + *A. lumbricoides* (3%, 95% CI: 1%–6%). In this review, intestinal parasitic infection was associated with handwashing after toilet (OR = 2.46, 95% CI: 1.11–5.45), handwashing before a meal (OR = 3.85, 95% CI: 1.71–8.68), the status of hand hygiene (OR = 2.99, 95% CI: 2–4.48), and untrimmed hand fingernails (OR = 2.11, 95% CI: 1.51–2.96).

**Conclusion:**

In Ethiopia, intestinal parasitic infections are prevalent among prisoners. The common parasitic infections were *E. histolytica/dispar* and *E. histolytica/dispar* + *H. worms.* Among prisoners, intestinal parasitic infection is associated with handwashing before a meal, handwashing after toilet, the status of hand hygiene, and untrimmed fingernails.

## 1. Introduction

In developing nations, the infection is primarily associated with inadequate personal and environmental cleanliness, contaminated food and drink, and inadequate toilet facilities. In tropical nations, schistosomiasis and soil‐transmitted helminthiasis are the most prevalent intestinal parasitic infections (IPIs) [1]. Geohelminths and enteric protozoa are present worldwide in varied degrees of prevalence and can be easily transmitted from person to person through the fecal‐oral route in a variety of circumstances. Soil‐transmitted helminthiasis infections were linked to an estimated 642.72 million cases and 1.38 million DALYs in 2021, with ascariasis accounting for 293.80 million cases, hookworm illnesses for 112.82 million cases, and trichuriasis for 266.87 million cases. An estimated 3472 deaths were linked to STH diseases, primarily ascariasis [2].

One of the populations highly vulnerable to intestinal parasite infestations is prisoners. The living conditions of prisoners in underdeveloped nations are characterized by inadequate facilities, malnourishment, limited access to drinkable water, overcrowding, and poor cleanliness [3]. In addition, prisoners are vulnerable to IPI since they have no control over their living conditions [4]. The risks are increased by inadequate healthcare facilities, weakened immunity from stress, high‐risk behaviors, and inadequate nutrition [5]. In poor nations, prisoners are ignored and do not benefit from health programs intended for regular citizens [6]. The 2024 World Prison Report stated that Ethiopia had the highest number of prisoners in East Africa [7]. The diverse mix of prisoners from various parts of Ethiopia may provide an ideal environment for the spread of various intestinal parasite species.

Research conducted in several prison facilities revealed a wide range in the prevalence of IPI. The prevalence of intestinal parasites in Malaysian prisons was approximately 26.5% [5]; prisoners in Midwest Brazil, 20.2% [8]; prisoners of Jos Central Prison, Nigeria, 14.4% [4]; prisoners in Kathmandu, Nepal, 6% [9]; prisoners in central jail of Bhopal, India, 6.7% [10]; and prisoners in Kenya, 24.7% [11]. In comparison to previous research, the prevalence of IPI was very high in Ethiopia. As report from study conducted in Bedele prison, the prevalence of IPI was 64% [12]; in Shewa Robit prison, it was 61.8% [13]; prisoners of Mekelle prison, it was 42.6% [14]; Arba Minch prison, southern Ethiopia, it was 48.1% [15]; in eastern Tigrai zonal prison, it was 40% [16]; and in Hosanna Town prison, it was 39.2% [17].

There is a previous related systematic review and meta‐analysis that was conducted to assess the global prevalence and determinants among prisoners, but the study does not show the burden and risk factors of IPI among Ethiopian prisoners [18]. In addition, the analysis did not take possible risk factors into account. Developing evidence on the risk factors and burden of IPI among prisoners is important for modifying the control strategy to account for prisons′ IPI control packages. Thus, to pool the prevalence estimates of IPI and variables associated with IPI in prisoners, we conducted this systematic review and meta‐analysis. The development of effective IPI control strategies and the guidance of further research depend on this information.

## 2. Methods

### 2.1. Protocol Registration

This study was not prospectively registered with the PROSPERO registry. The decision not to register was made due to the fact that this review synthesizes observational prevalence and risk factor data rather than comparing competing clinical interventions. Nonetheless, we adhered strictly to Preferred Reporting Items for Systematic Review and Meta‐Analysis (PRISMA) 2020 guidelines, documented all methods in this study, and made our database search strategies and data extraction forms fully transparent in the manuscript and Supporting Information.

### 2.2. Study Protocol and Reporting

This study was reported according to the PRISMA guidelines for study selection, literature search methods, data extraction, and result reporting (File S1) [19]. To find relevant data, we looked through gray literature sources and published studies that describe the prevalence and factors of IPI among prisoners and were published up until December 30, 2025. We looked through public databases such as PubMed, ScienceDirect, Google Scholar, and Semantic Scholar. Additionally, we reviewed the included papers′ reference lists to find any additional relevant studies that the database search might have overlooked. To find relevant articles, we used the following search strategy: ((“Prevalence” OR “magnitude” OR “burden”) AND (“intestinal parasitic infection” OR “Intestinal helminthiasis” OR “intestinal parasitosis” OR “soil transmitted helminthiases∗” OR “geohelminth∗”) AND (“associated factors” OR “determinants” OR “contributing factors” OR “risk factors”) AND (“prisoner” OR “inmates” OR “prisonprisoners” OR “Jail” OR “correctional facility”) AND (Ethiopia)).

Types of population: prisoners who reported having IPI.

Context: All studies conducted on the prevalence and determinants of IPI among prisonprisoners in Ethiopia were considered.

Condition: This review took into account studies that are relevant to the study objective, assessing the prevalence and determinants of IPI among prisonprisoners in Ethiopia.

### 2.3. Eligibility Criteria

#### 2.3.1. Inclusion Criteria

To develop the search phrases, the Condition, Context, and Population (COCOPO) principle was used. EndNote (Version X9) reference manager software was employed to evaluate, organize, and cite references. We included peer‐reviewed articles published in peer‐reviewed journals, as well as unpublished gray literature, including dissertations and theses. Studies written in any language with relevant data are part of this review. IPI infection in prisoners can be detected using any laboratory technique.

#### 2.3.2. Exclusion Criteria

Excluded studies included perspectives, case reports, case series, conference proceedings, systematic reviews, and meta‐analyses. Any research that did not fit the aforementioned eligibility requirements was removed once the full texts were reviewed.

### 2.4. Quality and Bias Assessment

Two authors, KM and DW, used the keywords to search Google Scholar, PubMed, Semantic Scholar, and ScienceDirect separately, and retrieved studies were assessed based on abstracts, titles, and full texts. The quality of the papers was assessed using the Joanna Briggs Institute′s (JBI) critical evaluation checklist for prevalence studies [20]. The sampling, sample size, sampling frame, representation, study setting description, study subjects, data analysis tools, nonresponse bias, reliability, and validity of the study tools are criteria used to evaluate relevant studies. These criteria were used to evaluate each study, and those that had a score of seven or above were incorporated in the study. On the other hand, the review did not include any studies that received a score of less than seven out of nine critical appraisal criteria for prevalence studies. Methodological quality assessment of included studies was performed individually by two different authors (AS and AM). Discrepancy between the two authors′ quality assessment was resolved by discussion with KM (File S2).

### 2.5. Study Selection and Data Extraction

Initially, after automatically eliminating duplicate studies, the titles and abstracts of the remaining studies were evaluated. Second, based on the evaluation of abstracts and titles, we excluded studies that were found to be irrelevant. Third, full‐text screening was performed independently by two reviewers (KM and DW), and those that were deemed ineligible were removed. Any disagreements at title, abstract, and full‐text screening were decided by discussion with a third reviewer (AS). The data extraction tool was created by DW and AM and reviewed by KM. This data extraction tool includes important information: the first author′s name, the study country, the year of publication, the study design, the sample size, the prevalence of IPI, *E. histolytica/dispar*, *A. lumbricoides*, *G. lamblia*, hookworms, *S. mansoni*, *Taenia* species, *E. coli*, *H. nana*, *T. trichiura*, *S. stercoralis*, and mixed infection.

### 2.6. Data Analysis

Data were extracted from pertinent primary sources using Microsoft Excel and exported to Stata software, Version 17, for data analysis. The data were visualized using forest plots, which also contained a 95% confidence interval to present the pooled effect size and weight of each included study [21, 22]. To pool the prevalence of IPI in prisoners in Ethiopia, a random‐effects meta‐analysis using the DerSimonian and Laird method was applied. To find out if there was statistical heterogeneity, the Cochran‐*Q* test and Higgins *I*‐squared (*I* ^2^) statistics were employed. When the associated *I*‐squared values were 25%, 50%, or 75%, heterogeneity was classified as low, moderate, or high [23]. A subgroup analysis was performed to investigate the possible causes of heterogeneity. To account for confounding variables, such as the year of publication, the number of positive cases, and the number of examined, we employed metaregression. Additionally, funnel plots were employed for visual presentation [24]. In a sensitivity analysis, the effect of a single study on the overall estimate was evaluated by gradually eliminating studies.

## 3. Results

**Figure 1:**
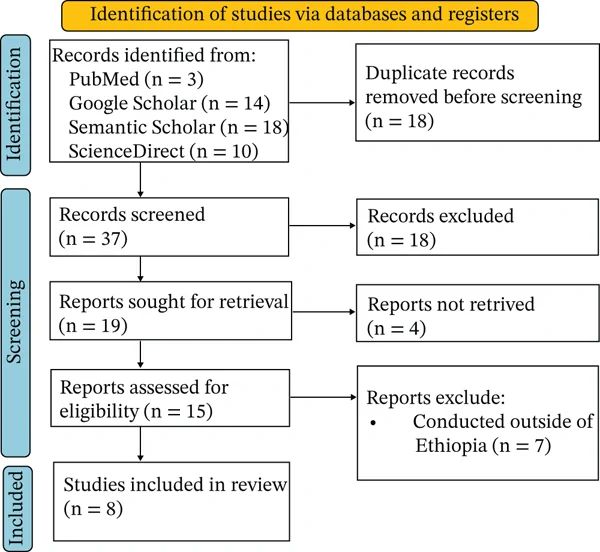
Flow diagram presenting the study selection process.

A total of 55 citations were found based on database searches on Google Scholar (n = 14), PubMed (n = 13), ScienceDirect (*n* = 10), and Semantic Scholar (*n* = 18), as illustrated in Figure 1. The remaining 37 papers were screened using their abstracts and titles after 18 duplicate entries were removed. Four entries were further removed because full texts could not be found, and the titles and abstracts of 18 of these studies were irrelevant to the study′s objective. For eligibility, the complete texts of the other 15 studies were carefully reviewed. Seven of these 15 studies were removed because they were conducted outside of Ethiopia. In the end, eight studies were included in this study.

### 3.1. Characteristics of Included Studies

This systematic review and meta‐analysis include eight suitable studies. The majority of the included studies are from central Ethiopia (25%, *n* = 2) [17, 25] and Northern Ethiopia (25%, *n* = 2, 14, 16), while the other studies come from four regions in Ethiopia. Specifically, one studies from north‐central Ethiopia [13], southern Ethiopia [15], Addis Ababa, Ethiopia [26], and southwestern Ethiopia [12](File S3).

### 3.2. Publication Bias Assessment

**Figure 2:**
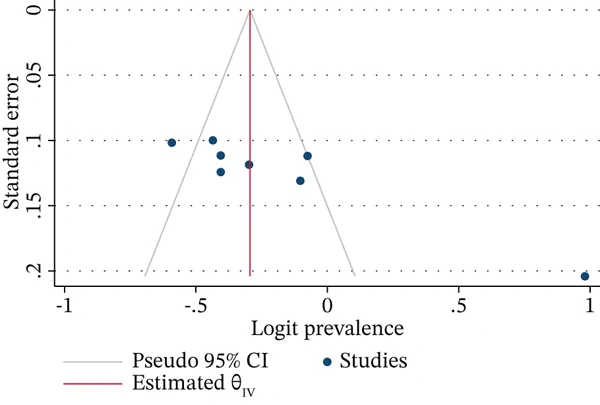
Graphical presentation of publication bias for prevalence of intestinal parasitic infection among prisoners in Ethiopia.

Publication bias was assessed qualitatively via visual inspection of funnel plots. Standard statistical testing was not carried out due to the unreliability of such tests when the included studies were small (*n* = 8). Visual inspection of the funnel plot revealed funnel plot asymmetry; there is evidence of publication bias (Figure 2). All studies passed through methodological quality assessment using the JBI critical evaluation checklist for prevalence studies to evaluate the risk of bias.

### 3.3. Metaregression Analysis

The fundamental causes of heterogeneity were identified by conducting metaregression. As a result, the year of publication, the number of examined, and the no. of positive cases were taken into account as covariates in a random‐effects metaregression. The metaregression analysis indicated that the number of examined and the no. of positive cases were significant predictors of the effect size (*p* < 0.05) (Table 1).

**Table 1:** Metaregression using the number of positive cases, year of publication, and the number of examined on the prevalence of intestinal parasitic infection among prisoners in Ethiopia.

Covariates	Coef	Std. Err	*Z*	*p* > |*z*|	[95% conf. interval]
Year of publication	−0.0100957	0.0507617	1.84	0.065	[−0.0058952, 0.1930869]
# of examined	−0.0100957	0.0026732	−3.78	0.000	[−0.0153352, −0.0048562]
No. of positive	0.6147832	0.2218008	2.77	0.006	[0.1800615, 1.049505]
Cons	−187.032	101.9027	−1.84	0.066	[−386.7575, 12.69357]

### 3.4. Sensitivity Analysis

**Figure 3:**
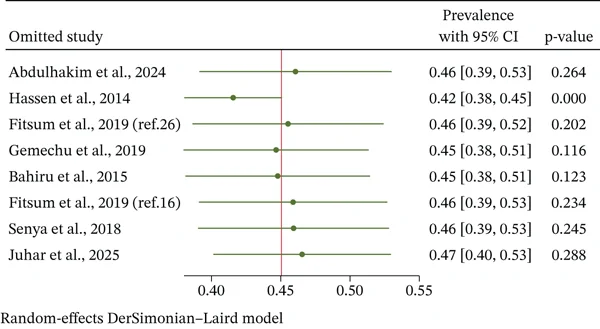
Leave‐one‐out sensitivity analysis.

The random‐effects model was used in a sensitivity analysis to determine the possible impact of a single study on the overall estimation. The leave‐one‐out sensitivity analysis demonstrated the robustness of our pooled estimate. Omitting any single study resulted in prevalence estimates ranging from 42% to 47%, all within the original confidence interval of 39%–51%. This confirms that no individual study disproportionately influenced our overall finding of 45% prevalence of IPI among prisoners in Ethiopia (Figure 3).

### 3.5. The Pooled Prevalence of IPI Among Prisoners in Ethiopia

**Figure 4:**
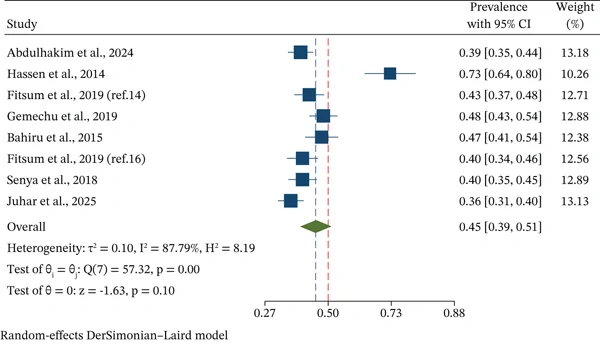
Forest plot of the overall prevalence of intestinal parasitic infection among prisoners in Ethiopia.

**Figure 5:**
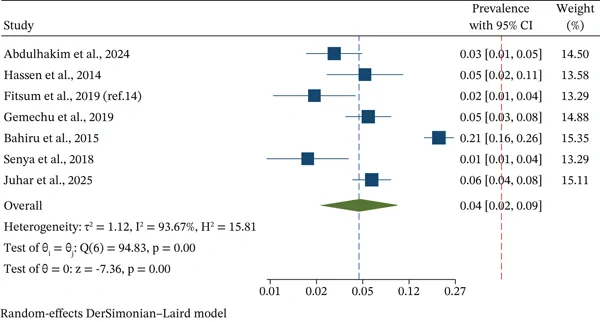
Forest plot of seven studies on the prevalence of hookworm infection among prisoners in Ethiopia.

**Figure 6:**
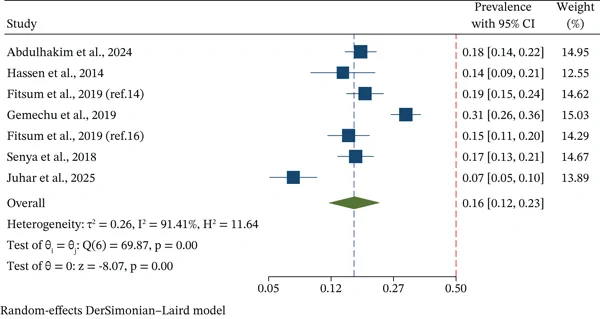
Forest plot of seven studies on the prevalence of *Entamoeba histolytica/dispar* infection among prisoners in Ethiopia.

**Figure 7:**
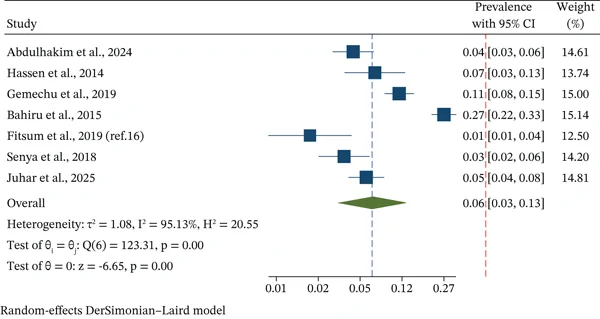
Forest plot of seven studies on the prevalence of *A. lumbricoides* infection among prisoners in Ethiopia.

**Figure 8:**
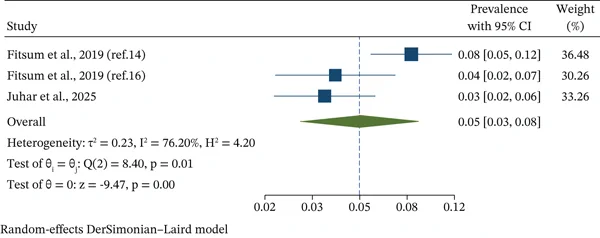
Pooled prevalence of *E. coli* infection among prisoners in Ethiopia.

**Figure 9:**
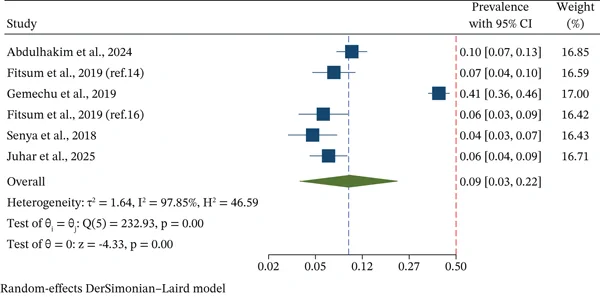
Forest plot of six studies on the prevalence of *Giardia lamblia* infection among prisoners in Ethiopia.

In this review, we have included eight studies involving 2412 prisoners in Ethiopia. The number of studies presenting species‐level prevalence of IPI among prisoners in Ethiopia included 87.5% (7/8) for *A. lumbricoides*, 87.5% (7/8) for hookworms, 87.5% (7/8) for *E. histolytica/dispar*, 75% (6/8) for *G. lamblia*, 37.5% (3/8) for *H. nana*, 37.5% (3/8) for *S. stercoralis*, 75% (6/8) for *Taenia* species, 37.5% (3/8) for *T. trichiura*, 37.5% (3/8) for *E. coli*, 37.5% (3/8) for *S. mansoni*, and 25% (2/8) for *E. vermicularis*According to a random‐effects model, the overall prevalence of IPI among prisoners in Ethiopia was 45% (95% CI: 39%–51%), with a high degree of heterogeneity (*I* ^2^ = 87.79*%*, *p* < 0.001) (Figure 4). The pooled species level pooled prevalence of intestinal parasitic infection was as follow: Hookworm 4% (95% CI: 2%–9%) (Figure 5), *E. histolytica/dispar* 16% (95% CI: 12%–23%) (Figure 6), *A. lumbricoides* 6% (95% CI: 3%–13%) (Figure 7), *E. coli* 5% (95% CI: 3%–8%) (Figure 8), and *G. lamblia* 9% (95% CI: 3%–22%) (Figure 9). Other less prevalent intestinal Parastic infections overall prevalence was *H. nana* 1% (95% CI: 1%–3%), *S. stercoralis* 4% (95% CI: 2%–8%), *T. trichiura* 3% (95% CI: 1%–9%), *Taenia species* 3% (95% CI: 2%–4%), and *S. mansoni* 1% (95% CI: 0%–2%) (File S4).

### 3.6. The Overall Prevalence of Multiple IPIs Among Prisoners in Ethiopia

**Figure 10:**
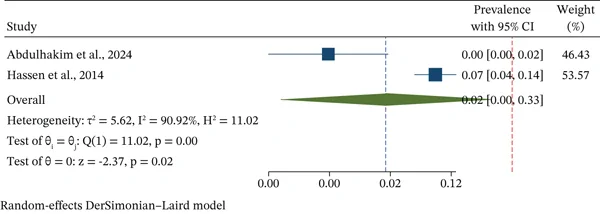
Pooled prevalence of *A. lumbricoides + H. worms* infection among prisoners in Ethiopia.

**Figure 11:**
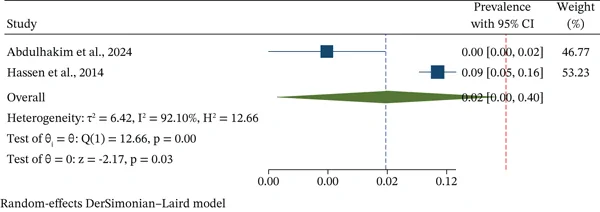
Pooled prevalence of *E. histolytica/dispar + H. worms* infection among prisoners in Ethiopia.

**Figure 12:**
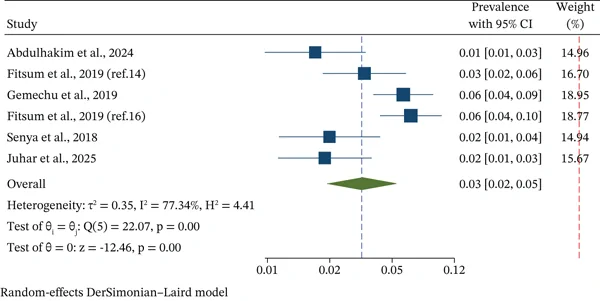
Forest plot showing pooled prevalence of *E. histolytica/dispar + G. lamblia* multiple infection among prisoners in Ethiopia.

**Figure 13:**
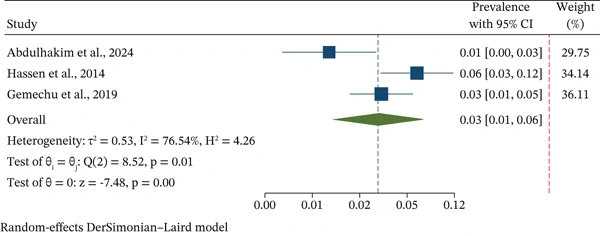
Forest plot showing pooled prevalence of *E. histolytica/dispar + A. lumbricoides* multiple infection among prisoners in Ethiopia.

The number of studies reporting the multiple IPI among prisoners in Ethiopia included 75% (6/8) for *E. histolytica/dispar* + *G. lamblia*, 25% (2/8) for *G. lamblia + Taenia* species, 25% (2/8) for *A. lumbricoides* + *H. worms*, 25% (2/8) for *E. histolytica/dispar* + *H. worms*, 37.5% (3/8) for *E. histolytica/dispar* + *A. lumbricoides*, 25% (2/8) for *E. histolytica/E. dispar* + *Taenia* species, 25% (2/8) for *E. histolytica/dispar* + *E. coli* + *G. lamblia*, and 37.5% (3/8) for *G. lamblia* + *E. coli*. Overall prevalence of multiple infections was *A. lumbricoides* + *H. worms* (2%, 95% CI: 0%–33%) (Figure 10), *E. histolytica/dispar* + *H. worms* (2%, 95% CI: 0%–4%) (Figure 11), *E. histolytica/dispar* + *G. lamblia* (3%, 95% CI: 2%–5%) (Figure 12), and *E. histolytica/dispar* + *A. lumbricoides* (3%, 95% CI: 1%–6%) (Figure 13). Other less common mixed infections were *E. histolytica/dispar* + *G. lamblia* + *E. coli* (2%, 95% CI: 0%–40%), *E. histolytica/E. dispar* + *Taenia* species (1%, 95% CI: 0%–6%), *G. lamblia* + *E. coli* (1%, 95% CI: 0%–3%), and *G. lamblia + Taenia* species (1%, 95% CI: 0%–2%) (File S4).

### 3.7. Subgroup Analysis by Regions

**Figure 14:**
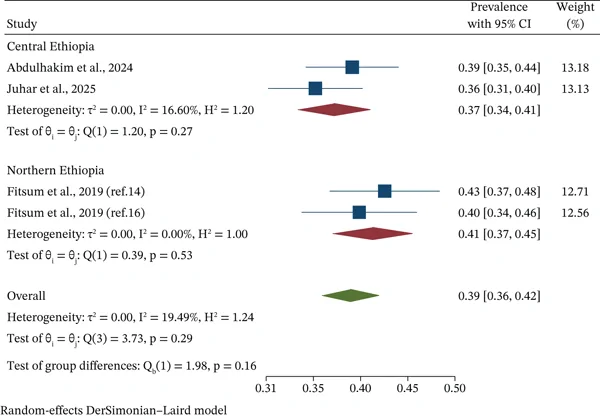
The pooled prevalence of IPI among prisoners in Ethiopia by the region of the study.

This review included studies conducted in six regions, including central Ethiopia, Northern Ethiopia, southern Ethiopia, Addis Ababa, Ethiopia, north‐central Ethiopia, and southwestern Ethiopia. However, in the subgroup analysis, we have considered regions with two or more studies. According to the regional subgroup analysis, the prevalence of IPI was in central Ethiopia (37% [95% CI: 34–41]) and Northern Ethiopia (41% [95% CI: 37–45]), but this difference is not statistically significant (*p* = 0.16) (Figure 14).

### 3.8. Subgroup Analysis Based on Publication Year and Sample Size

**Figure 15:**
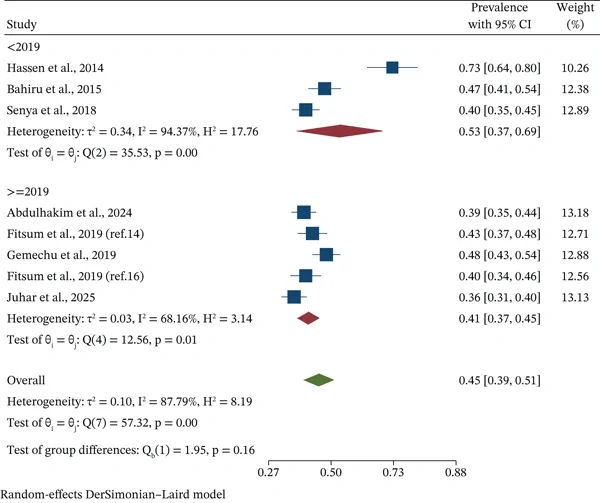
The pooled prevalence of IPI among prisoners in Ethiopia by the year of publication.

**Figure 16:**
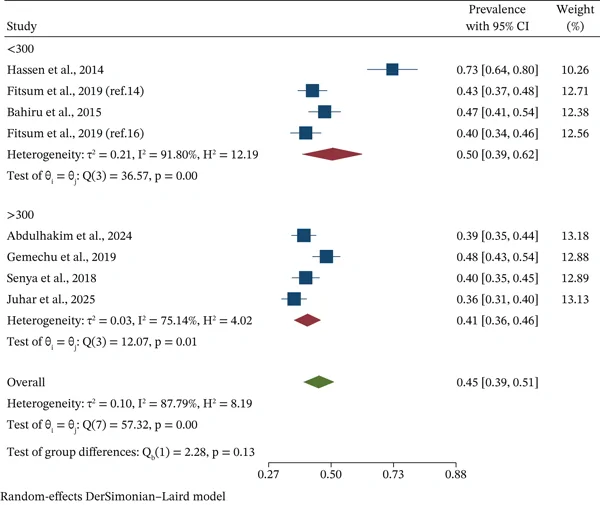
The pooled prevalence of IPI among prisoners in Ethiopia by sample size.

Regarding the study period, five studies were done after 2019, and the remaining three studies were done before 2019. In relation to sample size, individual studies were nearly equally distributed above and below 300 samples. Subgroup analysis by publication year showed no statistically significant difference in prevalence between studies published before 2019 (53%, 95% CI: 37–69) and those published from 2019 onwards (41%, 95% CI: 37–45) (*p* = 0.16) (Figure 15). Similarly, studies with sample sizes below 300 (50%, 95% CI: 39–62) did not differ significantly from those with larger samples (41%, 95% CI: 36–46) (*p* = 0.13) (Figure 16).

### 3.9. Risk Factors of IPI Among Prisoners in Ethiopia

The pooled risks among Ethiopian prisoners were assessed using studies involving at least two common significant risk factors. In this study, IPI was associated with handwashing before a meal, handwashing after using the toilet, the status of hand hygiene, and untrimmed fingernails. The pooled results show that failure to wash hands after toilet use increases the risk of IPI among prisoners in Ethiopia (OR = 2.46, 95% CI: 1.11–5.45). Accordingly, this pooled AOR (*I* ^2^ = 88.6*%*, *p* < 0.001) for handwashing after toilet use should be interpreted as exploratory rather than confirmatory. The results are presented to highlight potential associations that need further study in larger, homogeneous studies, and any recommendations based on the results should be made with due caution.

**Figure 17:**
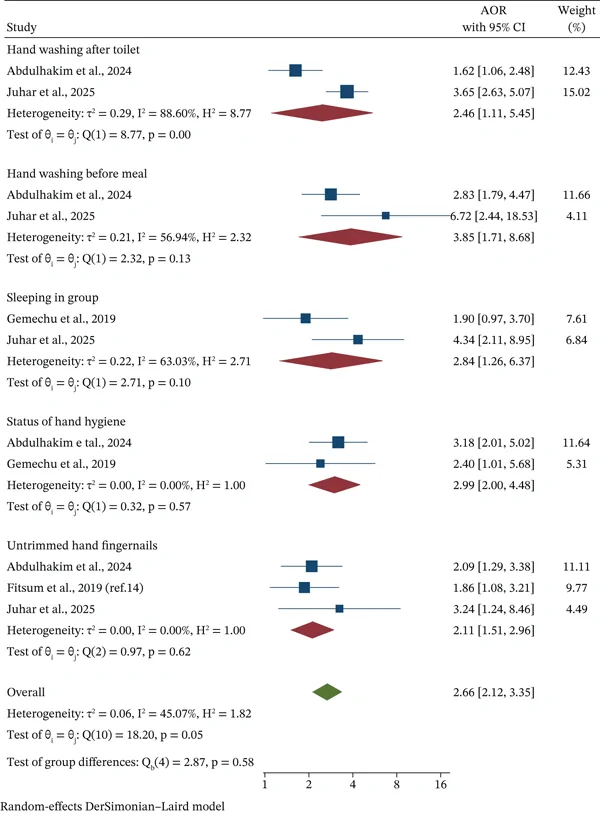
Forest plot presenting the overall significant risk factors associated with intestinal parasitic infection among prisoners in Ethiopia.

Similarly, prisoners who did not wash their hands before eating had 3.85 times higher odds of having an IPI than their counterparts (OR = 3.85, 95% CI: 1.71–8.68). Prisoners who sleep in groups had a higher chance of acquiring an intestinal parasitic infection compared with those who sleep individually OR = 2.84, 95% CI: 1.26–6.37). Prisoners who had unclean hands were 2.99 times more likely to have an IPI than those with clean hands (OR = 2.99, 95% CI: 2–4.48). Compared to prisoners with trimmed hand fingernails, those having untrimmed hand fingernails had 2.11 times higher odds of IPI (OR = 2.11, 95% CI: 1.51–2.96) (Figure 17).

## 4. Discussion

Globally, greater than 10.99 million people were imprisoned, and there were an estimated 113,727 prisoners between 2013 and 2014 in Ethiopia [7]. Prisoners in Ethiopia have inadequate housing and sanitary conditions, which are marked by overcrowding, poor medical care, poor hygienic conditions, insufficient food, and inaccessible sanitary supplies [27]. In addition, prisoners have no control over their living conditions, which puts them at risk for IPI [4]. These IPIs can cause malnutrition, anemia, persistent diarrhea, and abdominal pain, all of which are detrimental to one′s physical and mental well‐being [28, 29]. To the best of our knowledge, no prior meta‐analysis of the burden and risk of IPI among prisoners in Ethiopia has been carried out. This study provides data on the prevalence and determinants of IPI among prisoners in Ethiopia. Data from eight studies involving 2412 prisoners were included in this meta‐analysis. Thus, the results of this study could be useful in directing future research and creating efficient strategies to control the transmission and reduce the burden of parasitic infection.

This study showed that the pooled prevalence of IPI among prisoners in Ethiopia was 45%. However, this pooled estimate should be interpreted with care because this degree of heterogeneity suggests that the observed variation in prevalence across studies is not a result of chance but rather reflects actual differences in the study populations, study settings, sample size, and diagnostic method applied. In this study, the overall prevalence of IPI was greater than in study carried out in other country, prisoners in Midwest Brazil (20.2%) [8]; correctional facility, Jos, Nigeria (22.8%) [30]; Ajang Prison, Selangor, Malaysia (26.5%) [5]; Kisii prison, Kisii County, Kenya (24.7%) [11]; Rio de Janeiro (Brazil) (17.5%) [31]; central Ethiopia (35.6% and 39.2%) [17, 25]; Addis Ababa, Ethiopia (40%) [26]; and northern Ethiopia (40% and 42.6%) [14, 16]. In contrast, this finding is lower than studies conducted in Ebonyi State, southeastern Nigeria (76.29%) [32], north‐central Ethiopia (72.7%) [13], southern Ethiopia (48.1%) [15], and southwestern Ethiopia (47.4%) [12].

According to subgroup analysis, the highest reported pooled prevalence of IPI was reported from north‐central Ethiopia, published before 2019, and with a sample size of less than 300. The reported variation can arise from the true difference among the included studies, such as the diagnostic method used, sample size, and the number of samples examined. The high prevalence of IPI in publications before 2019 may have been caused by social and economic advancements in the country in general, particularly in prisons, as well as the success of national intestinal parasite prevention and control initiatives, which may have contributed to the recent decline in the burden of intestinal parasite infections. Moreover, in this study, subgroup analysis based on sample size shows that the lower sample size results lead to a higher prevalence of IPI.

In the present study, the most common intestinal parasite species were *E. histolytica* 16% (95% CI: 12%–23%), followed by *G. lamblia* 9% (95% CI: 3%–22%), *S. stercoralis* 4% (95% CI: 2%–8%), and hookworm 4% (95% CI: 2%–9%). The least often reported parasite species were *H. nana* (1%, 95% CI: 1%–3%) and *S. mansoni* (1%, 95% CI: 0%–2%). Other studies conducted in the region also reported the higher prevalence of *E. histolytica* and G*. lamblia* among prisoners [14, 15]. These protozoan cysts can survive for long periods of time outside of their hosts and are resistant to environmental conditions, which makes it easier for them to spread [33]. They are more common in places with inadequate sanitation and hygiene practices because they are frequently disseminated through tainted food or water.

In this study, we also present the pooled prevalence of mixed infection: *E. histolytica/dispar* + *G. lamblia* and *E. histolytica/dispar* + *A. lumbricoides* were the common multiple infections among prisoners. In line with our findings, other studies conducted in Ethiopia also reported the predominance of *E. histolytica/dispar* + *G. lamblia* [15, 16] and *E. histolytica/dispar* + *A. lumbricoides* coinfections [13, 15]. This finding is also supported by a study conducted in Nigeria reporting the predominance of E. *histolytica/dispar* + *H. worms* coinfection [32]. Geographical location, climate, and host‐related characteristics such as age‐related vulnerability, immunity, and exposure to polluted environments may all contribute to the variations in coinfection patterns.

IPI was generally strongly associated with inadequate handwashing habits. According to our findings, prisoners who did not wash their hands after defecating were more than two times more likely to contract a parasitic infection than those who did so regularly. This result is in line with several studies conducted in Ethiopia that identified infrequent handwashing after toilet use as a risk factor of IPI [34, 35]. Similarly, the current study also identified a hand with poor hand hygiene as a source of infection. This result is in line with findings from a study done in the south and central Ethiopia regions [15, 17].

In this study, prisoners who did not wash their hands before eating had a 3.85‐fold increased risk of developing an IPI. This result is consistent with research done in the Eastern Region of Nepal [36] and Indonesia [37]. It could be as a result of dust particles and the infective stage of parasites on unwashed hands, which facilitate the fecal‐oral transmission of microorganisms. In relation to sleeping status, prisoners who sleep in groups have an increased risk of IPI compared who sleep individually. The finding is supported by findings from studies conducted in central Ethiopia [25] and southern Ethiopia [15]. The main causes of this increased risk are sleeping in crowded living situations, leading to environmental contamination, which encourages the spread of parasites [15]. Therefore, it is crucial to address overcrowding of prisoners by improving prison infrastructure and administration.

According to this meta‐analysis, prisoners with untrimmed fingernails are more likely to have an IPI. Research conducted in several parts of Ethiopia also reported that prisoners who have untrimmed fingernails are more vulnerable to infections [15]. This is because it is difficult to maintain hygiene with untrimmed fingernails, and they can carry infectious stages of parasites and act as a reservoir for diseases. The high prevalence of *E. histolytica/dispar* and *G. lamblia* may be linked to the high proportion of untrimmed fingernails among prisoners [17]. This emphasizes how crucial it is to make sure prisoners have access to basic hygiene supplies and obtain education on personal hygiene as part of an infection prevention program.

## 5. Conclusion and Recommendations

IPI among prisoners is common in Ethiopia. Among the most common mono was *E. histolytica/dispar*, and multiple infections were *E. histolytica/dispar* + *A. lumbricoides* and *E. histolytica/dispar* + *G. lamblia*. Among prisoners, IPI is associated with handwashing before a meal, handwashing after toilet, the status of hand hygiene, and untrimmed fingernails. As a result, prison administrations, the Ministry of Health, and other stakeholders must supply suitable sanitary supplies, like soap and potable water, and encourage proper personal hygiene. Additionally, preventing and controlling the spread of parasites may depend on the implementation of periodic medical checkup programs and thorough health education centered on personal hygiene.

### 5.1. Limitations of the Study

All of the included studies in this review only collected stool samples once, even though a typical intestinal parasite diagnosis requires at least three samplings. Additionally, gold standard techniques for diagnosing certain parasites were not taken into account, which may have an impact on their prevalence. Additionally, every study that was included was cross‐sectional, which may share the drawbacks of a cross‐sectional study design.

## Author Contributions

Kemal Mohamed: formal analysis, conceptualization, investigation, software, methodology, visualization, writing—review and editing, writing–original draft, data curation, validation, supervision, and resources. Abdulhakim Mussema: data curation, conceptualization, investigation, visualization, supervision, validation, and writing—original draft. Asnake Simieneh: data curation, visualization, software, writing—review and editing, conceptualization, formal analysis, and methodology. Dagmawi Woldesenbet: conceptualization, methodology, formal analysis, validation, project administration, data curation, supervision, software, and writing—original draft. The authors listed under this work fulfill the minimum recognized requirements by making a substantial contribution to the research and are accountable for the work undertaken.

## Funding

No funding was received for this manuscript.

## Disclosure

All authors read and approved the final manuscript.

## Ethics Statement

Ethical approval and informed consent are not necessary because this is a systematic review of previously published works.

## Consent

The authors have nothing to report.

## Conflicts of Interest

The authors declare no conflicts of interest.

## Supporting Information

Additional supporting information can be found online in the Supporting Information section.

## Supporting information


**Supporting Information 1** File S1: PRISMA 2020 checklist.

**Supporting Information 2** File S2: The quality assessment using JBI standardized checklist of the studies included for review on burden and associated factors of intestinal parasitic infection among prisoners in Ethiopia: a systematic review and meta‐analysis.

**Supporting Information 3** File S3: Characteristics of included studies and prisoners in Ethiopia.

**Supporting Information 4** File S4: Figures showing the pooled prevalence of intestinal mono and multiple infections among prisoners in Ethiopia.

## Data Availability

The data supporting the study may be found in this article′s Supporting Information.
