# Supplementary material for: The Burden and Associated Factors of Intestinal Parasitic Infection Among Prisoners in Ethiopia: A Systematic Review and Meta‐Analysis
Source: J Parasitol Res. 2026 Sep 7;2026:7271924. doi: 10.1155/japr/7271924 (PMC13550994; doi:10.1155/japr/7271924)
Supplement: Supplementary file 2 [file JAPR-2026-7271924-s002.docx]

Figures showing the pooled prevalence of intestinal mono- and multiple infections among prisoners in Ethiopia.

Figure 1: Pooled prevalence of *H.nana* infection among prison inmates in Ethiopia.

Figure 2: Pooled prevalence of *S. stercoralis* infection among prison inmates in Ethiopia.

Figure 3: Pooled prevalence of *T. trichiura* infection among prison inmates in Ethiopia.

Figure 4:Forest plot of 6 studies on the magnitude of *Taenia species* infection among prison inmates in Ethiopia.

Figure 5: Pooled prevalence of *S. mansoni* infection among prison inmates in Ethiopia.

Figure 6: Pooled prevalence of *E. histolytica / dispar + E. coli + G. lamblia* infection among prison inmates in Ethiopia.

Figure 7: Pooled prevalence of *E.histolytica / E. dispar + Taenia species* infection among prison inmates in Ethiopia

Figure 8:Pooled prevalence of *G.lamblia+Taenia species* infection among prison inmates in Ethiopia

Figure 9: Forest plot showing pooled prevalence of *G. lamblia + E. coli* multiple infection among prison inmates in Ethiopia.
