# Supplementary material for: The Burden and Associated Factors of Intestinal Parasitic Infection Among Prisoners in Ethiopia: A Systematic Review and Meta‐Analysis
Source: J Parasitol Res. 2026 Sep 7;2026:7271924. doi: 10.1155/japr/7271924 (PMC13550994; doi:10.1155/japr/7271924)
Supplement: Supplementary file 3 [file JAPR-2026-7271924-s003.docx]

| **Table: Characteristics of included studies, prison inmates in Ethiopia** | | | | | | | | |
| --- | --- | --- | --- | --- | --- | --- | --- | --- |
| Author & Year of Publication | Abdulhakim etal.,2024(1) | Hassen etal,2014(2) | Fitsum etal,2019(3) | Gemechu etal.,2019(4) | Bahiru etal.,2015(5) | Mardu etal.,2019(6) | Juhar etal.,2025(7) | Senya etal,2018(8) |
| Country | Central Ethiopia | North-Central Ethiopia | Northern Ethiopia | Southern Ethiopia | Southwestern Ethiopia | Northern Ethiopia | Central Ethiopia | Addis Ababa  , Ethiopia |
| Study Design | Cross sectional | Cross sectional | Cross sectional | Cross sectional | Cross sectional | Cross sectional | Cross sectional | Cross sectional |
| No. of tested | 420 | 121 | 291 | 320 | 234 | 270 | 421 | 335 |
| No. of positive | 165 | 88 | 124 | 154 | 111 | 108 | 150 | 134 |
| *E.histolytica/dispar* | 75 | 17 | 55 | 99 |  | 41 | 29 | 56 |
| *E. coli* |  |  | 23 |  |  | 10 | 14 |  |
| *Giardia lamblia* | 41 |  | 20 | 131 |  | 15 | 26 | 15 |
| *Ascaris lumbricoides* | 17 | 8 |  | 36 | 63 | 4 | 23 | 11 |
| *Hookworms* | 11 | 6 | 5 | 17 | 48 |  | 24 | 5 |
| *T. trichiura* |  | 7 |  | 4 | 32 | 3 | 4 |  |
| *E. vermicularis* |  |  |  |  | 4 | 2 |  |  |
| *S. mansoni* | 2 |  |  |  |  | 1 | 6 |  |
| *S. stercoralis* |  | 5 |  | 19 |  |  |  | 5 |
| *H.nana* |  | 2 |  |  | 1 |  |  | 5 |
| *Taenia species* | 6 |  | 7 | 8 |  | 8 | 11 | 18 |
| *S. hematobium* |  |  |  | 7 |  |  |  |  |
| *E.histolytica/dispar+ G. lamblia* | 6 |  | 9 | 18 |  | 17 | 7 | 6 |
| *E.histolytica/dispar + E. coli* |  |  |  |  |  |  | 2 |  |
| *E.histolytica/dispar + E. coli + G. lamblia* |  |  |  |  |  | 2 | 3 |  |
| *E.histolytica/E.dispar+ E.vermicularis* |  |  | 1 |  |  |  |  |  |
| *E.histolytica/E.dispar + S.mansoni* |  |  | 1 |  |  |  |  |  |
| *E.histolytica/E.dispar + Taenia species* |  |  | 1 |  |  |  |  | 7 |
| *E.histolytica/dispar + A. lumbricoides* | 4 | 7 |  | 9 |  |  |  |  |
| *E.histolytica/diyspar + Hookworms* | 1 | 11 |  |  |  |  |  |  |
| *G. lamblia + E. coli* |  |  | 1 |  |  | 5 | 1 |  |
| *E.histolytica/E.dispar + T. trichiura + Hookworm* |  |  | 1 |  |  |  |  |  |
| *E.histolytica/diyspar + T. trichiura* |  | 8 |  |  |  |  |  |  |
| *A. lumbricoides + Hookworms* | 1 | 9 |  |  |  |  |  |  |
| *G. lamblia + Taenia species* | 1 |  |  | 3 |  |  |  |  |
| *G. lamblia + S. hematobium* |  |  |  | 4 |  |  |  |  |
| *Hookworm + Taenia species* |  | 5 |  |  |  |  |  |  |
| *S. stercoralis + T. trichiura* |  | 3 |  |  |  |  |  |  |
| *Hookworm + T. trichuris + S. stercoralis* |  |  |  | 1 |  |  |  |  |
| *Taeniaspecies*  *+ T. trichiura* |  |  |  |  |  |  |  | 6 |

**N.B: In the above table columns are not mutually exclusive**

**Reference**

1.	Mussema A, Tafesse W, Temam L. Prevalence of Intestinal Parasite Infection Among Prison Inmates and Their Associated Risk Factors at Hosanna Town, South–Central Ethiopia: A Cross‐Sectional Study. Journal of Parasitology Research. 2024;2024(1):7677743.

2.	Mamo H. Intestinal parasitic infections among prison inmates and tobacco farm workers in Shewa Robit, north-central Ethiopia. PLoS One. 2014;9(6):e99559.

3.	Mardu F, Yohannes M, Tadesse D. Prevalence of intestinal parasites and associated risk factors among inmates of Mekelle prison, Tigrai Region, Northern Ethiopia, 2017. BMC infectious diseases. 2019;19(1):406.

4.	Ameya G, Zerdo Z, Tesfaye M, Jabesa C, Awaje A, Dejene K, et al. Intestinal parasite infections and associated factors among inmates of Arba Minch prison, southern Ethiopia: cross sectional study. BMC infectious diseases. 2019;19(1):1086.

5.	Terefe B, Zemene E, Mohammed AE. Intestinal helminth infections among inmates in Bedele prison with emphasis on soil-transmitted helminths. BMC research notes. 2015;8(1):779.

6.	Mardu F, Berhe B, Tesfay K, Negash H. Assessment of sanitary condition of services as implication for intestinal parasitic infections among prison inmates: institutional based cross-sectional study in eastern Tigrai zonal prison, northern Ethiopia, 2018. BMC research notes. 2019;12(1):393.

7.	Nigus J, Lambiyo T, Dejen P. Prevalence and determinants of intestinal parasitic infections among prison inmates in Central Ethiopia. Tropical Diseases, Travel Medicine and Vaccines. 2025.

8.	Asfir S. Prevalence of Intestinal Parasites among Diarrhogenic Patients at Qilinto Prison Inmates of Addis Ababa, Ethiopia: Addis Ababa University; 2018.
