# Supplementary material for: The Burden and Associated Factors of Intestinal Parasitic Infection Among Prisoners in Ethiopia: A Systematic Review and Meta‐Analysis
Source: J Parasitol Res. 2026 Sep 7;2026:7271924. doi: 10.1155/japr/7271924 (PMC13550994; doi:10.1155/japr/7271924)
Supplement: Supplementary file 4 [file JAPR-2026-7271924-s004.docx]

**The quality assessment using the JBI standardized checklist of the studies included for review on burden and drivers of intestinal parasitic infection among inmate prisoners in Ethiopia: a systematic review and meta-analysis.**

| Author | Appropriateness of sampling frame | Appropriateness of sampling method | Adequate sample size | Describe the study setting and participants | Data analysis with sufficient coverage | Valid methods for condition identification | Valid and reliable outcome measurement | Appropriate statistical analysis | Adequate response rate | Study Level Bias Score |
| --- | --- | --- | --- | --- | --- | --- | --- | --- | --- | --- |
| Abdulhakim etal.,2024(1) | YES | YES | YES | YES | YES | YES | YES | YES | YES | 9/9=100% |
| Hassen etal,2014(2) | YES | YES | YES | YES | YES | YES | YES | YES | YES | 9/9=100% |
| Fitsum etal,2019(3) | YES | YES | YES | YES | YES | YES | YES | YES | YES | 9/9=100% |
| Gemechu etal.,2019(4) | YES | YES | YES | YES | YES | YES | YES | YES | YES | 9/9=100% |
| Bahiru etal.,2015(5) | YES | NO | YES | YES | YES | YES | YES | YES | YES | 8/9=88.9% |
| Mardu etal.,2019(6) | YES | YES | YES | YES | YES | YES | YES | YES | YES | 9/9=100% |
| Juhar etal.,2025(7) | YES | NO | YES | YES | YES | YES | YES | YES | YES | 8/9=88.9% |
| Senya etal,2018(8) | YES | NO | YES | YES | YES | YES | YES | YES | YES | 8/9=88.9% |

**Reference**

1.	Mussema A, Tafesse W, Temam LJJoPR. Prevalence of Intestinal Parasite Infection Among Prison Inmates and Their Associated Risk Factors at Hosanna Town, South–Central Ethiopia: A Cross‐Sectional Study. 2024;2024(1):7677743.

2.	Mamo HJPO. Intestinal parasitic infections among prison inmates and tobacco farm workers in Shewa Robit, north-central Ethiopia. 2014;9(6):e99559.

3.	Mardu F, Yohannes M, Tadesse DJBid. Prevalence of intestinal parasites and associated risk factors among inmates of Mekelle prison, Tigrai Region, Northern Ethiopia, 2017. 2019;19(1):406.

4.	Ameya G, Zerdo Z, Tesfaye M, Jabesa C, Awaje A, Dejene K, et al. Intestinal parasite infections and associated factors among inmates of Arba Minch prison, southern Ethiopia: cross sectional study. 2019;19(1):1086.

5.	Terefe B, Zemene E, Mohammed AEJBrn. Intestinal helminth infections among inmates in Bedele prison with emphasis on soil-transmitted helminths. 2015;8(1):779.

6.	Mardu F, Berhe B, Tesfay K, Negash HJBrn. Assessment of sanitary condition of services as implication for intestinal parasitic infections among prison inmates: institutional based cross-sectional study in eastern Tigrai zonal prison, northern Ethiopia, 2018. 2019;12(1):393.

7.	Nigus J, Lambiyo T, Dejen PJTD, Travel Medicine, Vaccines. Prevalence and determinants of intestinal parasitic infections among prison inmates in Central Ethiopia. 2025.

8.	Asfir S. Prevalence of Intestinal Parasites among Diarrhogenic Patients at Qilinto Prison Inmates of Addis Ababa, Ethiopia: Addis Ababa University; 2018.
